# 54478 The NIH Reporter Database: A Wealth of Information for Developing Team Science Metrics?

**DOI:** 10.1017/cts.2021.679

**Published:** 2021-03-30

**Authors:** Hosen Arman, Ziyad Razeq, Nancy Reichmann, Edmund Lattime, Biju Parekkadan

**Affiliations:** 1 Rutgers University Camden; 2 Rutgers Graduate School of Biomedical Sciences; 3 Rutgers Robert Wood Johnson Medical School; 4 Rutgers Cancer Institute of New Jersey; 5 Rutgers School of Engineering

## Abstract

ABSTRACT IMPACT: As scientific research is trending towards greater interdisciplinary and collaboration in order to meet the challenges of contemporary science, which has led to increased recognition of the importance of Team Science, this study will promote team science research within NJ ACTS Consortium as well as across the country. OBJECTIVES/GOALS: The objective of this study is to assess the feasibility of using the NIH Reporter database for developing and tracking team science metrics within the CTSA-funded NJ ACTS Consortium, which consists of RU, PU, and NJIT. The NIH Reporter database provides detailed information on single-PI and multiple-PI R01 grants funded by NIH. METHODS/STUDY POPULATION: 58 multi-PI projects and 344 single-PI projects are currently funded within the NJ ACTS consortium. We will use information from the database on funding levels, institutional composition of projects (e.g., within-consortium projects vs. projects with PIs both within and outside of the consortium), numbers of publications, impact factors of publications, and funding supplements obtained to quantify and track NIH R01 Team Science activity in the consortium. RESULTS/ANTICIPATED RESULTS: Preliminary analysis suggests that it will be both feasible and efficient to use the NIH reporter database to develop Team Science metrics and to augment information in the database with information on PI characteristics such as department/center/school/university, academic discipline, and rank/tenure status, as well and detailed composition of research teams, such as the mix in terms of senior and junior scholars. DISCUSSION/SIGNIFICANCE OF FINDINGS: This study will make an important contribution to this movement by demonstrating the feasibility of using the publicly available NIH Reporter Database to quantify the level and success of Team Science in the form of single-PI and multiple-PI R01 grants funded by NIH, which represent extremely important Team Science activities at universities.

